# Human respiratory syncytial virus and hospitalization in young children in Italy

**DOI:** 10.1186/s13052-018-0492-y

**Published:** 2018-05-04

**Authors:** Parvanè Kuhdari, Federica Brosio, Cristina Malaventura, Armando Stefanati, Andrea Orsi, Giancarlo Icardi, Giovanni Gabutti

**Affiliations:** 1 Department of Medical Sciences, Section of Public Health Medicine, University of Ferrara, via Fossato di Mortara 64/b, Ferrara, Italy; 2 Department of Medical Sciences, Section of Pediatrics, University of Ferrara, Ferrara, Italy; 3 Department of Health Sciences, University of Genoa, “Ospedale Policlinico San Martino IRCCS” teaching hospital, Genoa, Italy

**Keywords:** Human respiratory syncytial virus, Epidemiology, Impact, Hospitalization

## Abstract

**Background:**

Human respiratory syncytial virus (hRSV) is ubiquitous and causes respiratory diseases in both children and adults. Worldwide, hRSV pneumonia is the second cause of postnatal infant death after malaria. Given the high impact in terms of morbidity, mortality and costs, especially in the pediatric population, hRSV is recognized as a global health problem and the WHO, in view of the availability of new vaccines, has urged an active surveillance program of virus-related infections. The aim of this study has been to evaluate the impact of hRSV infections in the Italian population, particularly the pediatric one, in terms of hospitalizations.

**Methods:**

In the period 2001–2014**,** Hospital Discharge Records (HDRs) with the following diagnosis codes included in the primary diagnosis were evaluated: 466.11 (hRSV bronchiolitis), 480.1 (hRSV pneumonia) and 796 (hRSV). HDRs were supplied by the National Archive of HDRs data, Ministry of Health.

**Results:**

During the period 2001–2014, 57,656 hospital admissions due to hRSV pathologies were performed. Most hospitalizations (88.8%) involved patients with less than 1 year of age. Considering only primary diagnosis, 93% of the admissions were due to bronchiolitis, 5% to pneumonia and 2% to not otherwise specified hRSV infections. In the period 2001–2014, the hospitalization rate in 0–2 years old children, was equal to 224.8, 9.6 and 4.6/100,000 for hRSV bronchiolitis, hRSV pneumonia and not otherwise specified hRSV infection, respectively.

**Conclusions:**

This study confirms the high impact of hRSV on the pediatric population in the age class 0–4 years, with a peak in the first 12 months of life. Most hospitalizations were urgent, although the duration of the hospital stay was for the most part less than a week, with ordinary discharge at home. Pending the conclusion of ongoing clinical trials on different hRSV vaccine types, it is extremely important to have updated data on the impact of hRSV-related pathologies in the various age groups.

## Background

Human respiratory syncytial virus (hRSV), belonging to the Paramyxoviridae family, is ubiquitous and causes respiratory diseases in both children and adults [1].

In early childhood, it is the main etiologic agent of infections of the lower respiratory tract and is related to several clinical pictures such as bronchiolitis, pneumonia, ashmatic bronchitis. Bronchiolitis is the most common respiratory infection in < 1 year of age children and is the main cause of hospitalization in the youngest child, especially during the winter season [2].

hRSV infection can have a serious course especially in < 3 months of age children or in children with particular risk factors; it can require hospitalization in Intensive Care Unit and need for respiratory care [3]. The major risk factors for severe bronchiolitis caused by hRSV include preterm birth with or without chronic pulmonary disease, congenital heart disease, Down’s syndrome, neuromuscular disorders and immunodeficiencies [4]; the same risk factors are often related to a higher rate of hRSV-related hospitalization [5]. It has also been shown a correlation between the composition of the nasopharyngeal microbiota (in particular if this latter is characterized by *Haemophilus influenzae* and *Streptococcus)* and the severity of the disease [6].

In adults, instead, hRSV infection may, in the majority of cases, cause a mild inflammation of the upper respiratory tract or may be completely asymptomatic. Differently, in the elderly and in high risk adults (affected by chronic obstructive pulmonary disease, asthma, congestive heart failure, ect.), hRSV may cause a disease similar to that of non-pandemic influenza A [7].

In developed countries, pediatric mortality due to hRSV is low, but not nothing; the situation is completely different in developing countries, where the annual global number of deaths among < 5 years of age children is 66,000–199,000 and 28,000–111,500 for hRSV and influenza, respectively [8]. Worldwide, hRSV pneumonia is the second cause of postnatal infant death after malaria, causing 137,000 deaths each year (equal to 6.7% of all newborn deaths) [9].

The “RSV Observatory” study, has shown that hRSV infection in Italy presents a seasonal trend and, as described in other temperate countries, epidemics generally occur during the colder months of the year January–March) [10].

In the United States, from 1993 to 2008, a hospitalization rate due to hRSV infection equal to 55/100,000 person-years was recorded; this value is slightly lower than that for flu-related hospitalizations virus (65/100,000/year). In the < 2 years of age pediatric population these rates increase to 2345 admissions/100,000 and 151/100,000 person-years for hRSV and influenza, respectively [11]. These data show that, in the USA, every winter, hRSV is responsible for 1–2% of admission in < 2 years of age children. Quite similar data are recorded in Europe [12]. hRSV causes more hospital admissions than flu even in older children. In the USA, it is estimated that every year 1 out of 13 < 5 years of age children requires medical attention due to a hRSV infection [13].

In addition to ribavirin, other drugs are not currently licensed. However, transmission of the infection may be controlled by adequate hygienic measures and prophylaxis with Palivizumab [14]. The latter is a humanized monoclonal neutralizing antibody, produced by recombinant DNA technology, directed against the preserved epitope of the viral fusion glycoprotein, but its efficacy as well as costs are quite high [15–18]. Therefore, it is necessary to identify subjects at risk and to define the methods of administration to ensure a proper use of health resources [19].

Vaccination could be the most effective preventive measure, but, at the moment, different vaccine types are still under development [20–22].

Given the high impact in terms of morbidity, mortality and costs, especially in the pediatric population, hRSV is recognized as a global health problem and the World Health Organization (WHO), in view of the availability of new vaccines, has urged an active surveillance program of virus-related infections [23, 24].

Taking into account the unavailability of specific notification data for hRSV-related diseases and the possible future preventive opportunities with new vaccines, the purpose of this study has been to evaluate the impact of hRSV infections in the Italian population, particularly the pediatric one, in terms of hospitalizations in the period 2001–2014.

## Methods

The Hospital Discharge Record (HDR) was officially established in 1991 (Decree of the Ministry of Health of 28 December 1991) with the aim of providing a summary of the key information contained in the clinical record. HDRs represent the information gathering tool for each patient discharged by national public and private hospitals and include information on both clinical and organizational aspects of hospitalization. The coding of clinical information included in each HDR is done through the ICD9-CM coding system (International Classification of Disease, 9th Revision, Clinical Modification), currently used in Italy. For this retrospective study, HDRs were required for all hRSV-related pathologies for the period 2001–2014.

In detail, hospitalizations with the following ICD9-CM diagnosis codes included in the primary diagnosis were evaluated: 466.11 (hRSV bronchiolitis), 480.1 (hRSV pneumonia) and 796 (hRSV).

Data processing was performed through the Microsoft Excel 2007 Software and JMP for statistical analysis.

Resident population data for each given year (available on the National Statistics Institute, ISTAT, website) were used for calculating rates.

HDRs were supplied by the National Archive of HDRs data, Ministry of Health, General Directorate of Healthcare Planning, VI Office. For this type of retrospective study formal consent is not required; any personal data was protected accordingly to the Helsinki Declaration and to the Italian law (Legislative Decree of 30 June 2003, n. 196. Code on the protection of personal data).

## Results

From the analysis of the database provided by the Ministry of Health, it emerged that in Italy, during the period 2001–2014, 57,656 hospital admissions due to hRSV pathologies were performed. Males were more represented than females (55.1% vs. 44.9%, *p* < 0,001).

**Fig. 1:**
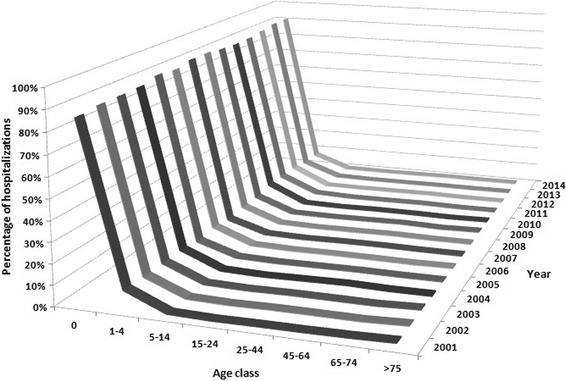
Hospitalization trend, stratified by age class and year of hospital admission

Most hospitalizations (88.8%) involved patients with less than 1 year of age; 8.2% of hospital admissions pertained to the 1–4 years age class. The distribution of hospitalizations remained substantially unchanged in the various examined years (Fig. 1). With regard to individual years, the trend of hospitalizations is characterized by a slightly increase over time, with periodic fluctuations and a peak in 2012, when 8.6% of all hospital admissions in the studied period were registered. The hospitalization procedure was mainly urgent (89.3% of cases) and the duration of stay was mainly (79.5% of cases) less than one week. Interestingly, duration of stay resulted independently associated with age class: the average hospitalization period increased from 5 days in children aged 0–14 years to 10 days in elderly aged > 74 years. The discharge method was mainly the ordinary one at home (96.4% of cases). In 0.1% of cases the patient died, 22.9% of which with less than 1 year of age and 58.4% aged > 64 years. The 1.7% of all hRSV-related hospitalizations registered in the period 2001–2014 involved infants (< 28 days of life). Lombardy, followed by Emilia-Romagna, was the region with the highest hospitalization rate: the distribution of hospitalizations due to hRSV was not uniform among regions, with a statistically significant prevalence (*p* < 0,001) of northern regions over central and southern ones, due to an average age of hospitalized subjects in northern regions lesser than that observed in central and southern regions.

**Fig. 2:**
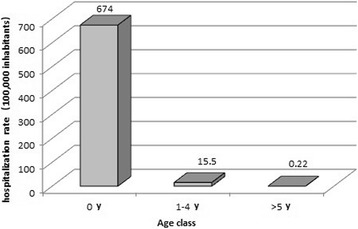
Hospitalization rate (per 100,000) stratified by age class

Figure 2 shows hospitalization rates by age group; the < 1 year age class is the one with the highest hospitalization rate (674/100,000 subjects).

**Fig. 3:**
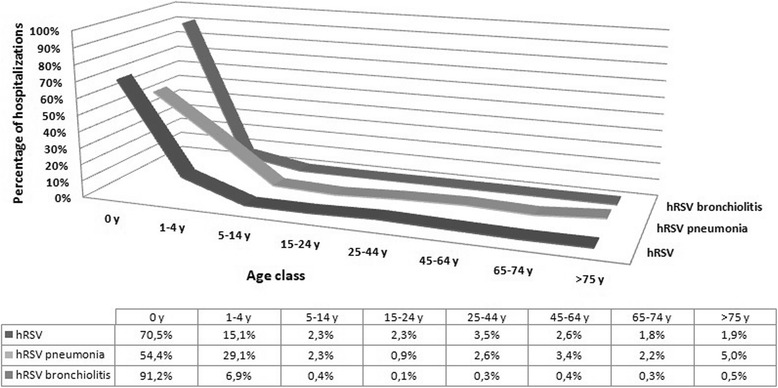
Hospitalizations stratified by diagnosis and age class, 2001–2014

Considering only primary diagnosis, 93% of the admissions were due to bronchiolitis, 5% to pneumonia and 2% to not otherwise specified hRSV infection. Figure 3 shows the distribution of hospitalization due to different diagnosis, stratified by age class.

Since the age most affected subjects were 0-1 and 2 years old children (bronchiolitis mainly affects children < 2 years of age), a more in-depth evaluation of this age group was performed. In the period 2001–2014, out of a total of 54,661 hospitalized children between 0 and 2 years, 93% were less than one year old and 23.5% of them were 2 months old.

The most represented diagnosis is hRSV bronchiolitis, even among younger children (94%). Considering the first year of life, the distribution of cases accordingly to different diagnosis per month of age shows the highest number of cases in the second month of life.

In the period 2001–2014, the hospitalization rate in 0–2 years old children, was equal to 224.8, 9.6 and 4.6/100,000 for hRSV bronchiolitis, hRSV pneumonia and not otherwise specified hRSV infection, respectively.

## Discussion

This study confirms the high impact of hRSV on the pediatric population in the age class 0–4 years, with a peak in the first 12 months of life. Most hospitalizations were urgent, although the duration of the hospital stay was for the most part less than a week, with ordinary discharge at home. Only 0.1% of the hospitalized patients died.

Despite being a public health problem worldwide, there are limited data on infant mortality for RSV. A recent systematic review showed that 99% of deaths concerned subjects from developing countries; the main risk factors in these children included: the presence of co-morbidities, especially congenital heart disease (28% of cases) and prematurity; the majority of died children received mechanical ventilation, which reflects the low level of quality of care in these countries. Moreover, intensive pediatrics was available only for 24% of children, and this further underlines that increase in child mortality is affected by the inadequate access to treatment in these countries [25].

In the period under review (2001–2014), the hospitalization rate was equal to 674/100,000 and to 15.5/100,000 inhabitants in the < 1 year and 1–4 years age classes, respectively. These results are in line with some published data for the most involved age groups even if other studies reported significant differences in hospitalization rates.

A retrospective survey on hRSV hospital admissions [26] was carried out in Sweden evaluating < 5 years of age children, who were hospitalized in 2004–2011. This survey exclusively enrolled children with a hRSV diagnosis confirmed by lab tests and showed that most hRSV pathologies affect children within the first year of life. The hospitalization rate was 17.4/1000 and 0.6/1000 in < 1 year and 1–4 years age children, respectively. A Spanish observational study on hRSV-related hospitalizations demonstrated a hospitalization rate equal to 2413/100,000 in < 2 years old children. An even higher hospitalization rate was found in infants, being equal to 4136/100,000; the average age of hospitalized subjects was about 5.8 months [27], confirming the greater impact of hRSV on < 2 years of age children, particularly on those < 6 months of age.

A study performed in England confirmed again that most hospitalization due to hRSV occur in the first months of life; the median age of hospitalized children was 3 months and a peak of hospitalizations was registered in the first month of life [28].

An Italian study evaluated the epidemiological characteristics of children hospitalized for bronchiolitis in ten consecutive seasons (2004–2014); viruses were identified in 48% of the samples and in 32.4% of the same hRSV was present, confirming its role in the pathogenesis of bronchiolitis [29]. Similarly, the relevant role of hRSV as the major etiologic agent of bronchiolitis was confirmed by two other retrospective studies. The first one was performed on < 12 months of life hospitalized children in 2004–2013 in a hospital in central Italy [30] and the other on < 15 years old children hospitalized for acute respiratory syndrome in the period 2008–2009; hRSV was identified in 31.3 and 34.1% of cases, respectively [31].

Similarly to this study, another retrospective research conducted in Spain [32] on hRSV bronchiolitis hospitalizations in < 1 year of age children between 2004 and 2012 confirmed an average lenght of stay of less than a week.

Compared to the above mentioned studies, hospitalization rates for hRSV and hRSV bronchiolitis in our study resulted much lower. The explanation for this inconsistency may be related to the lack of laboratory confirmation of the etiological diagnosis of infections. In Italy, hRSV diagnosis is mostly performed, on a clinical basis, without performing a direct diagnosis on nasopharyngeal swab. This probably implies a great underestimation of the pathology in hospitals; for the same reason, several hRSV-related diseases are not correctly classified (e.g. bronchiolitis by other viruses, ICD9-CM code 466.19).

In addition, in Italy the available studies that investigated the genetic diversity of circulating hRSV-A strains are limited, for example, in the period 1996–2006 [33], and in the 2010/11, 2011/12, and 2012/13 epidemic seasons [34].

hRSV vaccination could be the most effective means of prevention addressed to several subjects: pregnant women, newborns, < 6 months of age children, children of different ages (e.g. > 6 months to 2 years, 2–5 years and school children), > 60 years old subjects and immunocompromised people [9, 35]. There are several types of vaccine under development: live attenuated, subunit, and vectored vaccines [20]. The preventive approach through vaccination could be a useful tool especially for the extreme age classes, such as infants and the elderly, when the immaturity of the immune system in the first case or the immunosenescence in the second can predispose to a serious hRSV infection [36]. The development of the hRSV vaccine has been hampered by the “enhanced respiratory disease” (ERD) following natural infection in naïve children previously vaccinated with the formalin inactivated vaccine; ERD was probably related to the deposition of lung immune complexes and/or an imbalance of the immune response, with a Th2-biased response [37]. Ideally, a hRSV vaccine should stimulate mucosal immunity, activate the Th1 response to promote virus clearance and prevent ERD, and induce the production of neutralizing antibodies [37].

The attenuated live vaccine would offer some benefits, especially in younger children: it does not cause vaccine-associated disease, stimulates a broad immune response (innate and acquired, humoral and cellular, at systemic and respiratory level), could be administered intranasally (without need for puncture) replicating in the upper respiratory tract of young children despite the presence of neutralizing maternal antibodies [20, 38].

While traditional vaccines mainly induce neutralizing antibodies to protect against pathogens with a complex life cycle or high genetic instability, in this case, it is also crucial to activate the cell-mediated adaptive immune response (T lymphocytes, in particular cytotoxic CD8 + T lymphocytes). In the case of hRSV, some experimental studies have shown that while the presence of neutralizing antibodies protects only partially from severe pathology [39], the availability of pre-existing CD8 + T lymphocytes at intraepithelial level is related to a lesser severity of the pathology [40]; in other words, there is the need to stimulate both humoral and cellular immune response.

Genetic vaccines are able to induce this type of immune response. In such vaccines, the gene corresponding to the antigen of interest (transgene) is inserted into the genome of “vectors”, such as recombinant viruses. Among the different classes of viruses evaluated as carriers for genetic vaccines, Adenoviruses have shown to have a high-efficiency ability to infect mammalian cells, avoiding any integration into the human genome and stimulating, in addition to humoral and cell-mediated immune response, also the innate response through the pathogens-associated molecular patterns (PAMPs). Also in the case of the hRSV vaccine, vaccines based on recombinant vectors could induce the production of mRNA and antigenic proteins within guest cells as well as a humoral and cellular response. Various hRSV genes are candidates for this approach, in particular the gene for proteins F, G, N and M2–1 [41].

Vaccines based on subunits proteins would most likely evoke an Th2-biased response and be characterized by a pulmonary infiltrate. Genetic vaccines, on the other hand, have a lower risk of inducing vaccine-associated pathology because they behave like a live virus, producing antigens at intracellular level and activating a Th1 immune response [42].

The new European Medicines Agency (EMA) guidelines [43] address the development of RSV vaccines focusing on the assessment of their safety and efficacy in subjects at high risk of developing a lower respiratory tract infection or a severe hRSV disease, as well as the opportunity of immunization of pregnant women. Similarly to other vaccinations already recommended for pregnant women (flu vaccine and dTap) it is expected that women will be vaccinated in the third trimester to maximize the amount of maternal antibody transferred to the foetus. Dose regimen selection for pregnant women may be based on maximizing the difference in neutralising antibody titres in cord blood between infants born to vaccinated and unvaccinated mothers whilst maintaining an acceptable safety profile.

Pending the conclusion of ongoing clinical trials on different hRSV vaccine types, it is extremely important to have updated data on the impact of hRSV-related pathologies in the various age groups. Certainly the frequent lack of laboratory diagnosis implies a significant underdiagnosis/underreporting rate, and this also has an impact on the evaluation of hRSV-related hospitalizations. Even if hospital admissions represent only a part, albeit the most clinically relevant, of the total of hRSV infections, the data from this study confirm the relevant impact of hRSV, especially in the neonatal and pediatric age, in our country.

This observational study is the only survey that evaluates the impact of hRSV-related pathologies in terms of hospitalization in the Italian population. The study also covers a very large time frame (2001–2014). However, it evaluates hRSV-related hospitalizations (hRSV bronchiolitis, hRSV and hRSV pneumonia) referring only to the main diagnosis inserted in the HDRs. No hospitalizations have been considered where hRSV-related pathologies has been included as secondary diagnosis in HDRs and this could result in an underestimation of cases, particularly of those with respiratory failure as primary diagnosis and hRSV-bronchiolitis as secondary diagnosis.

## Conclusions

This study highlights that the impact of hRSV on the pediatric population in the age class 0–4 years is high, particularly in the first year of life, however, RSV also affects the elderly population, in particular in southern Italy. In the evaluated period (2001–2014), the hospitalization rate was equal to 674/100,000 and to 15.5/100,000 inhabitants in the < 1 year and 1–4 years age classes, respectively. These results are in line with other published data and contribute to confirm that hRSV is a global health issue. For this reason as well as for the promising development of new vaccines, the WHO has called for an active surveillance program of hRSV-related infections.
